# Operational efficiency evaluation of urban and rural residents' basic pension insurance system by utilizing a picture fuzzy TOPSIS method based on the cumulative prospect theory

**DOI:** 10.3389/fpubh.2022.1009207

**Published:** 2022-10-03

**Authors:** Yifan Xiong

**Affiliations:** School of Finance, Shanghai University of Finance and Economics, Shanghai, China

**Keywords:** multi-attribute group decision-making (MAGDM), picture fuzzy sets (PFSs), TOPSIS method, cumulative prospect theory (CPT), operational efficiency evaluation

## Abstract

Population aging is the most serious challenge facing the pension insurance system in China in the next few decades. Compared with the employees of civil servants and enterprises and institutions, urban and rural residents are unstable vulnerable groups with less income. In order to deal with the pension risks caused by the growing aging population and solve the security problems of urban and rural residents, our government has carried out a series of constructive works in the pension insurance system: in view of the rural and urban residents, new rural social endowment insurance system and the social endowment insurance system for urban residents have been set up and combined into a unified basic old-age insurance system for urban and rural residents in 2014. With the continuous expansion in the scale of income and expenditure of urban and rural living insurance funds and the size of the insured number, it is of great necessity to evaluate the efficiency of this system. The operational efficiency evaluation of urban and rural residents' basic pension insurance systems is viewed as multi-attribute group decision-making (MAGDM). In this paper, we propose an approach by combining the traditional Technique for Order Preference by Similarity to an Ideal Solution (TOPSIS) with cumulative prospect theory (CPT) which can be widely used with vague information. Thus, the main contribution of this study is as follows: (1) the TOPSIS method is extended by picture fuzzy sets (PFSs) with unknown weight information; (2) entropy method to obtain the original weights of attributes; (3) the picture fuzzy-CPT-TOPSIS (PF-CPT-TOPSIS) method is used to deal with the MAGDM problems under PFSs; (4) a numerical instance for operational efficiency evaluation of urban and rural residents' basic pension insurance systems is proposed to testify the effectiveness of new method; and (5) some comparative studies are provided to give effect to the rationality of PF-CPT-TOPSIS approach.

## Introduction

Data constitute the most important part in the procedure of selecting cooperators. However, we usually face some fuzzy information which increases the difficulty of turning information into numbers (1–6). For example, what a beautiful scenery. We cannot translate the degree of beauty into data, which leads to the loss of a mass of information. In order to solve the shortage, Zadeh (7) proposed the notion of fuzzy sets to describe vague information. In the procedure of decision-making, we usually invite some experts to evaluate alternatives and companies (8–12). In the process of evaluation, the psychological factor of the decision maker is also a very important factor that affects the decision result (13–16). Tversky and Kahneman (17) proposed the CPT to describe the psychology of decision-makers (DMs) that can improve the precision of data, and we utilize the idea to make up for the deficiency of the TOPSIS method in considering DMs' psychological factors.

At present, the trend of population aging in Chinese is becoming more and more obvious, and the problem of old-age care for the elderly has become a very serious social problem in China. At present, the Chinese old-age insurance system consists of four parts: enterprise supplementary old-age insurance, basic old-age insurance, personal savings old-age insurance, and commercial old-age insurance. Among them, the basic old-age insurance system combining social pooling and individual accounts is a new type of basic old-age insurance system pioneered by the Chinese in the world. It is an important part of the Chinese social security system. However, compared with employees of government agencies and public institutions and employees of urban enterprises, urban and rural residents do not have fixed work units and employers, and the tripartite financing model of the traditional social insurance system is not suitable for this group. However, with the intensification of the Chinese population aging trend, the urban and rural residents' basic endowment insurance system is under increasing pressure. In addition, due to the differences in the level of economic development in different regions of China, the operation status of urban and rural residents' basic endowment insurance also shows huge differences in different regions. Therefore, it is of great significance to evaluate the operational efficiency of the basic endowment insurance for urban and rural residents in various provinces and cities in China, and find out the existing problems to ensure the sustainable operation of the basic endowment insurance system for urban and rural residents and to provide scientific guidance for government departments to make decisions. The operational efficiency evaluation of urban and rural residents' basic pension insurance systems is viewed as multiple attribute decision-making (MADM). In this paper, we propose an approach by combining the traditional TOPSIS with CPT, which can be widely used with vague information. Thus, the motivation and innovative value of this study are as follows: (1) the TOPSIS method is extended by PFSs with unknown weight information; (2) entropy method to obtain the original weights of attributes; (3) the PF-CPT-TOPSIS method is used to deal with the MAGDM problems under PFSs; (4) a numerical instance for operational efficiency evaluation of urban and rural residents' basic pension insurance systems is proposed to testify the effectiveness of new method; and (5) some comparative studies are provided to give effect to the rationality of PF-CPT-TOPSIS approach. We divided the manuscript into six parts to describe the novel approach. Part 2 mainly introduces the works of some scholars on the TOPSIS method and the CPT aspect. In section 3, we mainly introduce some basic concepts about PFSs. Section 4 mainly describes the PF-CPT-TOPSIS approach. In section 5, a numerical instance for operational efficiency evaluation of urban and rural residents' basic pension insurance systems is proposed to testify the effectiveness of the new method. In order to check the accuracy of the method, we compare the overcome of a new method with the results of other available methods. Ultimately, we draw a conclusion in Section 7.

## Literature review

In recent years, dozens of scholars paid attention to researching fuzzy decision-making. The notion of fuzzy sets was proposed originally by Zadeh (7) in 1965. This concept had laid a solid foundation for the research of fuzzy decision-making. Aktaş and Çagman (18) defined the soft sets and soft groups. Maji et al. (19) gave an application of soft sets in a decision-making problem. Majumdar and Samanta (20) defined the generalized fuzzy soft sets. Maji (21) defined the intuitionistic fuzzy soft sets. Zhang (22) defined bipolar cognitive modeling and multiagent decision analysis. Zhang (23) defined the Yinyang bipolar fuzzy sets and fuzzy equilibrium relations. Abdullah et al. (24) defined the bipolar fuzzy soft sets. Ramot et al. (25) defined the complex fuzzy sets. Gulistan, Yaqoob, Elmoasry and Alebraheem (26) defined the complex bipolar fuzzy sets. Mahmood et al. (27) defined bipolar complex fuzzy Hamacher aggregation operators. Mahmood et al. (28) defined the bipolar complex fuzzy soft sets. On the basis of fuzzy sets, Atanassov (29) defined the concept of institution fuzzy sets (IFSs) that are composed of positive and negative membership degrees. Thereafter, Cuong and Kreinovich (30) proposed the notion of PFSs. Compared to intuitionistic fuzzy sets (IFSs), the PFSs have a more degree of neutral membership degree. Through it, we can describe the fuzzy information in detail. In order to distinguish some PFSs which have high similarity, Singh (31) put forward a novel way and testified its practicability by an instance. In the area of picture fuzzy (PF) operators, Garg (32) proposed a number of operators (PF hybrid average aggregation operator, etc.). Li et al. (33) extended the original Evaluation Based on Distance from Average Solution (EDAS) approach with PFSs. Then, they checked the validity of the new approach by a numerical instance. For the sake of overcoming the change in selecting a suitable sharing station, Lin et al. (34) built an extended MULTIMOORA (Multiobjective Optimization by Ratio Analysis plus full Multiplicative form) model on the basis of the Borda rule and a new score function in PFNs. Meksavang et al. (35) combined VIKOR (VIsekriterijumska optimizacija i KOmpromisno Resenje) method with picture fuzzy distance operators to tackle the problem of the selection of suppliers. Qiyas et al. (36) proposed a variety of operators with PFSs (such as picture fuzzy Yager weighted average) and utilized them to propose a novel approach. Wang et al. (37) extended some picture fuzzy operators and applied them to tackling MAGDM problems. Wang et al. (38) proposed a novel method that took the discrepancy of two PFNs and accommodation of two kinds of criteria. In the area of similarity measurement, Wei (39) proposed a novel form of the similarity measurement method and used it in strategic decisions. Wei (40) proposed some picture fuzzy aggregation operators and utilized them to develop some approaches to tackle MADM questions. Wei (41) extended the original TODIM (an acronym in Portuguese for Interactive Multi-criteria Decision-Making) approach with PFSs.

Lai et al. (42) proposed the TOPSIS approach. Chen et al. (43) took advantage of the original TOPSIS approach to tackle the issue of evaluating suppliers. Yong (44) made a choice of sites by using the TOPSIS method with triangle fuzzy numbers. Xu and Zhang (45) utilized the TOPSIS approach with hesitant fuzzy sets to select energy policy. Jahanshahloo et al. (46) presented an approach by combining TOPSIS with interval data. Jahanshahloo et al. (47) tackled an instance by utilizing the TOPSIS approach and triangle fuzzy numbers to describe fuzzy information. Wang et al. (48) proposed fuzzy hierarchical TOPSIS which can be more precise in terms of the conversion of guideline values to numbers. Arora and Naithani (49) proposed the TOPSIS method for MADM in computing exponential divergence measures under Pythagorean fuzzy sets. Petrovic and Kankaras (50) proposed the hybridized TOPSIS approach for air traffic control radar position selection. Yildirim et al. (51) proposed the robust Mahalanobis Distance based TOPSIS method for evaluating the economic development of provinces. Bozanic et al. (52) presented a specific methodology for the development of a neuro-fuzzy system, which is defined through a TOPSIS MADM method.

## Preliminary knowledge

In this section, we retrospect some fundamental knowledge about PFSs.

### Picture fuzzy sets

Cuong and Kreinovich (30) proposed the concept of PFSs.

**Definition 1** (30). Suppose that *D* is a non-empty set, PFSs can be ruled by Eq (1).

(1) $$\begin{array}{l} \Gamma =\left\{\langle \zeta ,j_{D}\left(\zeta \right),k_{D}\left(\zeta \right),l_{D}\left(\zeta \right)|\zeta \in D\rangle \right\} \end{array}$$

where the functions *j*_*D*_(ζ), *k*_*D*_(ζ), and *l*_*D*_(ζ) represent membership, neutral, and non-membership degree of the element ζ ∈ *D* in the set , respectively. PFSs must meet the requirement: . In addition, there is a refusal membership degree *w*_*D*_(ζ) which should meet the requirement: *j*_*D*_(ζ) + *k*_*D*_(ζ) + *l*_*D*_(ζ) + *w*_*D*_(ζ) = 1.

**Definition 2** (35). Suppose that there are two PFNs (picture fuzzy numbers): *F* = (*j*_*F*_(ζ), *k*_*F*_(ζ), *l*_*F*_(ζ)) and *G* = (*j*_*G*_(ζ), *k*_*G*_(ζ), *l*_*G*_(ζ)), then the algorithms of PFNs is reduced to (β > 0):

1. *F* ⊕ *G* = (*j*_*F*_(ζ) + *j*_*G*_(ζ) − *j*_*F*_(ζ)*j*_*G*_(ζ), *k*_*F*_(ζ)*k*_*G*_(ζ), *l*_*F*_(ζ)*l*_*G*_(ζ))
2. *F* ⊗ *G* = (*j*_*F*_(ζ)*j*_*G*_(ζ), *k*_*F*_(ζ) + *k*_*G*_(ζ) − *k*_*F*_(ζ)*k*_*G*_(ζ), *l*_*F*_(ζ) + *l*_*G*_(ζ) − *l*_*F*_(ζ)*l*_*G*_(ζ))
3. $\beta F=\left(1-{\left(1-j_{F}\left(\zeta \right)\right)}^{\beta },k_{F}^{\beta }\left(\zeta \right),l_{F}^{\beta }\left(\zeta \right)\right)$
4. $F^{\beta }=\left(j_{F}^{\beta }\left(\zeta \right),1-{\left(1-k_{F}\left(\zeta \right)\right)}^{\beta },1-{\left(1-l_{F}\left(\zeta \right)\right)}^{\beta }\right)$
5. $\bar{F}=\left(l_{F}\left(\zeta \right),k_{F}\left(\zeta \right),j_{F}\left(\zeta \right)\right)$

**Definition 3** (41). If there are two PFNs, the algorithms of score function *H* and accuracy function *J* that are benefit for us to tackle PFNs are shown as follows:

$$\begin{array}{l} \begin{array}{c} H_{F}\left(\zeta \right)=\frac{1+j_{F}\left(\zeta \right)-l_{F}\left(\zeta \right)}{2},H_{G}\left(\zeta \right)=\frac{1+j_{G}\left(\zeta \right)-l_{G}\left(\zeta \right)}{2}\;H_{F}\left(\zeta \right),H_{G}\left(\zeta \right)\in \left[0,1\right] \end{array} \end{array}$$

*J*_*F*_(ζ) = *j*_*F*_(ζ) + *k*_*F*_(ζ) + *l*_*F*_(ζ), *J*_*G*_(ζ) = *j*_*G*_(ζ) + *k*_*G*_(ζ) + *l*_*G*_(ζ)*J*_*F*_(ζ), *J*_*G*_(ζ) ∈ [0, 1]

**Definition 4** (41). We can use the numbers of *H* and to compare PFNs, and the rules of comparison are reduced to:

1. If *H*_*F*_(ζ) > *H*_*G*_(ζ), then *F* > *G*;
2. If *H*_*F*_(ζ) < *H*_*G*_(ζ), then *F* < *G*;
3. If *H*_*F*_(ζ) = *H*_*G*_(ζ), then

- If *J*_*F*_(ζ) > *J*_*G*_(ζ), then *F* > *G*;
- If *J*_*F*_(ζ) < *J*_*G*_(ζ), then *F* < *G*;
- If *J*_*F*_(ζ) = *J*_*G*_(ζ), then *F* = *G*.

**Definition 5** (38). We can utilize the function of Hamming distance to calculate the distance of two diverse PFNs, and the function is described as Eq (2).

(2) $$\begin{array}{l} e\left(F,G\right)=\frac{1}{5}\left(\begin{array}{c} \left|j_{F}-j_{G}|+|k_{F}-k_{G}|+|l_{F}-l_{G}\right|\\ +|\max\left\{j_{F},k_{G}\right\}-\max\left\{j_{G},k_{F}\right\}|\\ +|\max\left\{j_{F},l_{G}\right\}-\max\left\{j_{G},l_{F}\right\}| \end{array}\right) \end{array}$$

Except that, the Euclidean distance also has the same effect which is shown as Eq (3).

(3) $$\begin{array}{l} e^{*}\left(F,G\right)=\sqrt{\frac{1}{5}\left(\begin{array}{c} {\left(j_{F}-j_{G}\right)}^{2}+{\left(k_{F}-k_{G}\right)}^{2}+{\left(l_{F}-l_{G}\right)}^{2}\\ +{\left(\max\left\{j_{F},k_{G}\right\}-\max\left\{j_{G},k_{F}\right\}\right)}^{2}\\ +{\left(\max\left\{j_{F},l_{G}\right\}-\max\left\{j_{G},l_{F}\right\}\right)}^{2} \end{array}\right)} \end{array}$$

**Definition 6** (37). There are some algorithms, such as PFWA (picture fuzzy weighted average) and PFWG (picture fuzzy weighted geometric) operators, that can be applied to a series of PFNs, and the PFWA operator is reduced to:


$$\begin{array}{l} PFWA_{u}\left(K_{1},K_{2},...,K_{t}\right)=\overset{y}{\underset{t=1}{⊕}}\left(u_{t}K_{t}\right) \end{array}$$

For the rest, the PFWG operator can be shown as Eq (4).


(4) $$\begin{array}{l} PFWG_{u}\left(K_{1},K_{2},...,K_{t}\right)=\overset{y}{\underset{t=1}{⊗}}{\left(K_{t}\right)}^{u_{t}} \end{array}$$

where stands for the weight vector of *K*_*t*_(*t* = 1, 2, …, *y*) that should meet the requirement of $u_{t}\geq 0,\left(t=1,2,...,y\right),\overset{y}{\underset{t=1}{\sum }}u_{t}=1$.

**Theorem 1** (37). According to **Definition 2**, the PFWA operator is shown as Eq (5).

(5) $$\begin{array}{l} \begin{array}{l} PFWA_{u}\left(K_{1},K_{2},...,K_{t}\right)=\overset{y}{\underset{t=1}{⊕}}\left(u_{t}K_{t}\right)\\ =\left(1-\overset{y}{\underset{t=1}{\prod }}{\left(1-j_{K_{t}}\right)}^{u_{t}},\overset{y}{\underset{t=1}{\prod }}{\left(k_{K_{t}}\right)}^{u_{t}},\overset{y}{\underset{t=1}{\prod }}{\left(l_{K_{t}}+k_{K_{t}}\right)}^{u_{t}}-\overset{y}{\underset{t=1}{\prod }}{\left(k_{K_{t}}\right)}^{u_{t}}\right) \end{array} \end{array}$$

**Theorem 2** (37). Similarly, According to **Definition 2**, the PFWG operator is shown as Eq (6).

(6) $$\begin{array}{l} \begin{array}{l} PFWG_{u}\left(K_{1},K_{2},...,K_{t}\right)=\overset{y}{\underset{t=1}{⊗}}{\left(K_{t}\right)}^{u_{t}}\\ =\left(\overset{y}{\underset{t=1}{\prod }}{\left(j_{K_{t}}+k_{K_{t}}\right)}^{u_{t}}-\overset{y}{\underset{t=1}{\prod }}{\left(k_{K_{t}}\right)}^{u_{t}},\overset{y}{\underset{t=1}{\prod }}{\left(k_{K_{t}}\right)}^{u_{t}},1-\overset{y}{\underset{t=1}{\prod }}{\left(1-l_{K_{t}}\right)}^{u_{t}}\right) \end{array} \end{array}$$

where $u={\left(u_{1},u_{2},...,u_{t}\right)}^{T}$ stands for the weight vector of that should meet the requirement of $u_{t}\geq 0,\left(t=1,2,...,y\right),\overset{y}{\underset{t=1}{\sum }}u_{t}=1$.

## PF-CPT-TOPSIS model for MAGDM

**Step 1**. We can obtain the original matrices $A^{\alpha }={\left({\text{s}}_{df}^{\alpha }\right)}_{g\times h}$ which are obtained from DMs and are composed of PFNs. The matrix includes some fuzzy information, such as the degree of recognition of DMs and the uncertainty of DMs and so on. And the matrix *A*^α^ is shown as follows:

(7) $$\begin{array}{l} A^{\alpha }={\left(s_{df}^{\alpha }\right)}_{g\times h}=\left(\begin{array}{ccc} s_{11}^{\alpha } & \ldots & s_{1h}^{\alpha }\\ \vdots & \ddots & \vdots \\ s_{g1}^{\alpha } & \cdots & s_{gh}^{\alpha } \end{array}\right) \end{array}$$

**Step 2**. Normalize these PFNs by exchanging the positions of membership degree and non-membership degree in cost attributes.

(8) $$\begin{array}{l} {\tilde{s}}_{df}^{\alpha }=\left({\tilde{j}}_{df}^{\alpha },{\tilde{k}}_{df}^{\alpha },{\tilde{l}}_{df}^{\alpha }\right)=\{\begin{array}{cc} \left(j_{df}^{\alpha },k_{df}^{\alpha },l_{df}^{\alpha }\right) & \text{for benefit attribute};\\ \left(l_{df}^{\alpha },k_{df}^{\alpha },j_{df}^{\alpha }\right) & \text{for cost attribute}. \end{array} \end{array}$$

**Step 3**. After normalizing, we should integrate the normalized matrices by utilizing PFWA operators.

(9) $$\begin{array}{l} s_{df}=\overset{q}{\underset{\alpha =1}{⊕}}\left({\tilde{s}}_{df}^{\alpha }\right)=\\ \left(1-\overset{q}{\underset{\alpha =1}{\prod }}{\left(1-{\tilde{j}}_{df}^{\alpha }\right)}^{Q_{\alpha }},\overset{q}{\underset{\alpha =1}{\prod }}{\left({\tilde{k}}_{df}^{\alpha }\right)}^{Q_{\alpha }},\overset{q}{\underset{\alpha =1}{\prod }}{\left({\tilde{l}}_{df}^{\alpha }+{\tilde{k}}_{df}^{\alpha }\right)}^{Q_{\alpha }}-\overset{q}{\underset{\alpha =1}{\prod }}{\left({\tilde{k}}_{df}^{\alpha }\right)}^{Q_{\alpha }}\right) \end{array}$$

**Step 4**. Take advantage of entropy method to obtain the original weights of attributes, and the steps of entropy method are reduced to:

(1) Use Eq (10) to obtain the average value of *f*th attribute.

(10) $$\begin{array}{l} {\bar{s}}_{f}=\frac{\overset{g}{\underset{d=1}{\sum }}s_{df}}{g}=\left(\frac{\overset{g}{\underset{d=1}{\sum }}{\tilde{j}}_{df}}{g},\frac{\overset{g}{\underset{d=1}{\sum }}{\tilde{k}}_{df}}{g},\frac{\overset{g}{\underset{d=1}{\sum }}{\tilde{l}}_{df}}{g}\right)=\\ \left({\bar{j}}_{f},{\bar{k}}_{f},{\bar{l}}_{f}\right) \end{array}$$

where ${\bar{j}}_{df}$, ${\bar{k}}_{df}$, and ${\bar{l}}_{df}$ represent the average value of positive, neutral, and negative membership degree of *f*th attribute, separately.

(2) Calculate the Hamming distance $e\left({\tilde{s}}_{df},{\bar{s}}_{f}\right)$ between the average value of *f*th attribute and each PFN of *d*th alternative through utilizing Eq (11).

(11) $$\begin{array}{l} e\left({\tilde{s}}_{df},{\bar{s}}_{f}\right)=\frac{1}{5}\left(\begin{array}{c} \left|{\tilde{j}}_{df}-{\bar{j}}_{f}|+|{\tilde{k}}_{df}-{\bar{k}}_{f}|+|{\tilde{l}}_{df}-{\bar{l}}_{f}\right|\\ +|\max\left\{{\tilde{j}}_{df},{\bar{k}}_{f}\right\}-\max\left\{{\tilde{k}}_{df},{\bar{j}}_{f}\right\}|\\ +|\max\left\{{\tilde{j}}_{df},{\bar{l}}_{f}\right\}-\max\left\{{\tilde{l}}_{df},{\bar{j}}_{f}\right\}| \end{array}\right) \end{array}$$

(3) In order to find the original weights, we should obtain the entropy of the *f*th attribute (χ_*f*_) which can be computed by using Eq (12).

(12) $$\begin{array}{l} \chi_{f}=-\frac{1}{\ln\;g}\overset{g}{\underset{d=1}{\sum }}\left[\frac{e\left({\tilde{s}}_{df},{\bar{s}}_{f}\right)}{\overset{g}{\underset{d=1}{\sum }}e\left({\tilde{s}}_{df},{\bar{s}}_{f}\right)}\ln\;\left(\frac{e\left({\tilde{s}}_{df},{\bar{s}}_{f}\right)}{\overset{g}{\underset{d=1}{\sum }}e\left({\tilde{s}}_{df},{\bar{s}}_{f}\right)}\right)\right] \end{array}$$

(4) After computing χ_*f*_, we can acquire the original weights of χ_*f*_ attribute by Eq (13).

(13) $$\begin{array}{l} i_{f}=\frac{1-\chi_{f}}{\overset{g}{\underset{d=1}{\sum }}\left(1-\chi_{f}\right)},d=1,2,...,g \end{array}$$

**Step 5**. In traditional TOPSIS method, we take the positive and negative ideal points as reference point to measure the advantages and disadvantages of each alternative. We note the positive ideal point as $s^{+}=\left(s_{1}^{+},s_{2}^{+},...,s_{h}^{+}\right)$ and the negative ideal point as that can be found by utilizing Eq (14).

(14) $$\begin{array}{l} \{\begin{array}{c} s_{f}^{+}=\underset{d}{\max}s_{df}\\ s_{f}^{-}=\underset{d}{\min}s_{df} \end{array} \end{array}$$

**Step 6**. Figure out the number $L_{d}^{+}$ and $L_{d}^{-}$ of the prospect value of CPT, which represent the combined gains and losses of DMs facing gains and losses. The algorism is described as Eq (15–17).

(15) $$\begin{array}{l} \{\begin{array}{c} L_{d}^{+}=\overset{o}{\underset{f=1}{\sum }}m_{df}^{+}n^{+}\left(s_{f}\right)\\ L_{d}^{-}=\overset{h}{\underset{f=o+1}{\sum }}m_{df}^{-}n^{-}\left(s_{f}\right) \end{array} \end{array}$$

(16) $$\begin{array}{l} \{\begin{array}{c} n^{+}\left(s_{f}\right)=e{\left(s_{df},s_{f}^{-}\right)}^{b}\\ n^{-}\left(s_{f}\right)=-ve{\left(s_{df},s_{f}^{+}\right)}^{c} \end{array} \end{array}$$

(17) $$\begin{array}{l} \{\begin{array}{c} m_{f}^{+}=\frac{{\left(\overset{h}{\underset{f=1}{\sum }}i_{f}\right)}^{x}}{{\left({\left(\overset{h}{\underset{f=1}{\sum }}i_{f}\right)}^{x}+{\left(1-\overset{h}{\underset{f=1}{\sum }}i_{f}\right)}^{x}\right)}^{\frac{1}{x}}}-\frac{{\left(\overset{h}{\underset{f=o+1}{\sum }}i_{f}\right)}^{x}}{{\left({\left(\overset{h}{\underset{f=o+1}{\sum }}i_{f}\right)}^{x}+{\left(1-\overset{h}{\underset{f=o+1}{\sum }}i_{f}\right)}^{x}\right)}^{\frac{1}{x}}}\\ m_{f}^{-}=\frac{{\left(\overset{h}{\underset{f=1}{\sum }}i_{f}\right)}^{z}}{{\left({\left(\overset{h}{\underset{f=1}{\sum }}i_{f}\right)}^{z}+{\left(1-\overset{h}{\underset{f=1}{\sum }}i_{f}\right)}^{z}\right)}^{\frac{1}{z}}}-\frac{{\left(\overset{h}{\underset{f=o+1}{\sum }}i_{f}\right)}^{z}}{{\left({\left(\overset{h}{\underset{f=o+1}{\sum }}i_{f}\right)}^{z}+{\left(1-\overset{h}{\underset{f=o+1}{\sum }}i_{f}\right)}^{z}\right)}^{\frac{1}{z}}} \end{array} \end{array}$$

where $n^{+}\left(s_{f}\right)$, represent value function. $m_{f}^{+}$ and $m_{f}^{-}$ represent income decision weight and loss decision weight, respectively. In addition, the parameters indicate DMs' risk attitudes when they gain or loss, respectively. is the parameter that stands for loss aversion. Beyond that, the parameters of *x* and *z* describe the curvature of weighting function (). These parameters are proposed by Tversky and Kahneman (17).

**Step 7**. Then we extend the final step of traditional TOPSIS method to calculate the closeness coefficient δ_*d*_ of each alternative, and the function can be reduced to Eq (18).


(18) $$\begin{array}{l} \delta_{d}=\frac{\left|L_{d}^{+}\right|}{\underset{d}{\max}|L_{d}^{+}|}-\frac{\left|L_{d}^{-}\right|}{\underset{d}{\min}|L_{d}^{-}|} \end{array}$$

**Step 8**. After obtaining the number of δ_*d*_, we can acquire the ranking of alternatives in ascending order. The larger the number, the better the scheme.

## Numerical instance

Population aging is the most severe challenge facing any country's pension insurance system in the next few decades. Population aging refers to the process in which the elderly population continues to increase, and the proportion of the population gradually increases. Internationally, the proportion of the population over 60 years old in the total population is usually 10%, or the proportion of the population over 65 years old in the total population is 7%, which is the standard for a country or region to enter an aging society. According to the 2016 Social Service Development Statistical Bulletin published on the website of the Ministry of Civil Affairs, by the end of 2016, there were 231 million elderly people aged 60 years and above, accounting for 16.7% of the total population, of which 150 million were aged 65 years and above. It accounts for 10.8% of the total population, far exceeding the international standard of an aging society. In addition, Chinese actual national conditions have further aggravated the seriousness of the pension problem: on the one hand, the family planning policy implemented in China in the 1970 and 1980 s caused the growth rate of the young and middle-aged population to slow down, and the family structure became smaller and smaller. On the other hand, with the rapid growth of the economic level, people's living standards and health awareness have also improved, and the average life expectancy has increased, and the gap with developed countries is getting smaller and smaller. In addition, with Chinese industrialization and with the acceleration of urbanization, the social and economic structure is constantly changing, and the traditional security functions of family pensions and land pensions are gradually weakening. In the face of such a huge elderly population base and the increasingly severe degree of aging in our country, solving their pension problems has become an important task in the process of social development in our country. Since the real pension insurance system appeared in Germany in the 1870 and 1880 s, it has been quickly imitated by other countries in the world. The construction of the Chinese old-age insurance system has gone through a stage from the development idea of “emphasizing the city over the countryside” and the institutional arrangement of “separating groups of people” to the stage of the overall development of urban and rural areas. In the 1950 s, the Chinese modern old-age security system began. Due to the limitations in objective conditions, this stage was mainly aimed at the staff of state enterprises, public institutions, and collective institutions. After entering the new century, the scope of coverage was gradually expanded to urban and rural residents, embarking on the road of coordinated urban and rural development of the old-age insurance system. Compared with civil servants, employees of public institutions and enterprises, and urban and rural residents belong to the disadvantaged group with a less and unstable income, and they have greater expectations for “support for old age.” In order to cope with the pension risks brought about by the ever-increasing population aging, and to solve the problem of urban and rural residents getting old and providing support, the Chinese government has carried out a lot of constructive work in the design of the old-age insurance system. Social endowment insurance (hereinafter referred to as “new rural insurance”) and social endowment insurance for urban residents (hereinafter referred to as “urban home insurance”) were introduced, and by the end of 2012, the goal of full coverage of the above two systems has been basically achieved. Due to the convergence of the new rural insurance and urban residential insurance in terms of financing mode, payment level, payment level, subsidy method, etc., in 2014, the State Council decided to merge these two systems to establish unified nationwide urban and rural residents, that is, basic endowment insurance (hereinafter referred to as “urban and rural residential insurance”). With the combined implementation of the two systems, the pension insurance safety net with the largest number of participants and the widest range of benefits in the world is formed. According to the “2017 National Economic and Social Development Statistical Bulletin” issued by the National Bureau of Statistics, as of the end of 2017, the number of urban and rural residents covered by insurance was 513 million, of which more than 153 million received benefits. The establishment of a unified urban and rural residential insurance is of great practical significance for narrowing the gap between urban and rural areas and between regions, enabling urban and rural residents to equally enjoy basic old-age security and promoting the integration of urban and rural social security, which is embodied in the following two aspects: first, urban and rural residential insurance. The lack of government investment in the “Old Rural Insurance” has been changed, and it is clearly stipulated that the funds come from individual contributions, collective subsidies, and government subsidies, and the financial responsibility of the government in it is clarified from the system. Second, it is conducive to the realization of the goal of equalization of public services. The public services provided by the government to citizens should be roughly equal. The establishment of a unified urban and rural residential insurance has narrowed the differences between the two systems before the merger and expanded the coverage of the systems, which is conducive to the gradual realization of the equalization of basic public services targets. However, the operational efficiency evaluation of urban and rural residents' basic pension insurance systems can be attributed to the MAGDM problem. In this given section, we provide a real numerical example for operational efficiency evaluation of urban and rural residents' basic pension insurance systems through PF-CPT-TOPSIS method. Assume that five urban and rural residents' basic pension insurance systems *Q*_*i*_(*i* = 1, 2, 3, 4, 5) were chosen and four attributes were assessed for these urban and rural residents' basic pension insurance systems. Except that, in order to obtain a professional and suitable partner among them, the managers invite four experts s who come from the university. The weights of each experts are shown as: *R*_*E*_1__ = 0.2, *R*_*E*_2__ = 0.3, *R*_*E*_3__ = 0.3, *R*_*E*_4__ = 0.2. The six attributes are described as follows: ①W_1_ is basic pension fund income; ②W_2_ is the accumulated balance of basic pension insurance fund; ③W_3_ is the management of basic pensions; ④W_4_ is basic pension benefits; ⑤W_5_ is basic pension fund expenditure; and ⑥W_6_ is the salary expenditure of basic pension insurance fund managers. The five possible urban and rural residents' basic pension insurance systems *Q*_*i*_(*i* = 1, 2, 3, 4, 5) are to be evaluated by PFNs under the six given attributes. Among all attributes, *W*_5_ and *W*_6_ are cost attributes and other attributes are benefit attributes. After experts rate five companies, we can obtain four matrices with PFNs. The overcomes are presented in Tables 1–4. After experts rate five urban and rural residents' basic pension insurance systems, we can obtain four matrices with PFNs, and the overcomes are presented in Tables 1–4.

**Table 1:** Decision matrix given by the first expert *E*_1_.

	***W*_1_**	***W*_2_**	***W*_3_**	***W*_4_**	***W*_5_**	***W*_6_**
***Q*_1_**	(0.75, 0.04, 0.11)	(0.73, 0.06, 0.13)	(0.77, 0.05, 0.12)	(0.74, 0.09, 0.1)	(0.13, 0.07, 0.78)	(0.11, 0.07, 0.8)
***Q*_2_**	(0.81, 0.02, 0.1)	(0.85, 0.06, 0.07)	(0.79, 0.02, 0.01)	(0.7, 0.05, 0.07)	(0.05, 0.11, 0.81)	(0.04, 0.03, 0.8)
***Q*_3_**	(0.79, 0.06, 0.1)	(0.74, 0.07, 0.11)	(0.8, 0.12, 0.06)	(0.82, 0.07, 0.09)	(0.14, 0.05, 0.75)	(0.13, 0.11, 0.7)
***Q*_4_**	(0.77, 0.12, 0.09)	(0.81, 0.09, 0.1)	(0.78, 0.13, 0.09)	(0.72, 0.1, 0.13)	(0.11, 0.11, 0.75)	(0.1, 0.12, 0.71)
***Q*_5_**	(0.72, 0.11, 0.09)	(0.73, 0.08, 0.04)	(0.69, 0.15, 0.11)	(0.77, 0.09, 0.11)	(0.14, 0.07, 0.71)	(0.15, 0.11, 0.71)

**Table 2:** Decision matrix given by the second expert *E*_2_.

***W*_1_**	***W*_2_**	***W*_3_**	***W*_4_**	***W*_5_**	***W*_6_**	***W*_7_**
***Q*_1_**	(0.77, 0.12, 0.09)	(0.72, 0.08, 0.14)	(0.74, 0.12, 0.11)	(0.8, 0.09, 0.11)	(0.08, 0.09, 0.78)	(0.14, 0.09, 0.73)
***Q*_2_**	(0.81, 0.03, 0.06)	(0.84, 0.12, 0.03)	(0.76, 0.07, 0.1)	(0.73, 0.11, 0.05)	(0.09, 0.12, 0.79)	(0.1, 0.09, 0.8)
***Q*_3_**	(0.76, 0.13, 0.1)	(0.74, 0.12, 0.12)	(0.75, 0.09, 0.12)	(0.8, 0.07, 0.12)	(0.14, 0.07, 0.73)	(0.09, 0.08, 0.75)
***Q*_4_**	(0.78, 0.12, 0.09)	(0.75, 0.03, 0.11)	(0.74, 0.06, 0.12)	(0.73, 0.08, 0.13)	(0.13, 0.08, 0.77)	(0.12, 0.07, 0.71)
***Q*_5_**	(0.75, 0.1, 0.12)	(0.72, 0.08, 0.16)	(0.72, 0.12, 0.09)	(0.76, 0.13, 0.11)	(0.12, 0.05, 0.79)	(0.06, 0.09, 0.77)

**Table 3:** Decision matrix given by the third expert *E*_3_.

***W*_1_**	***W*_2_**	***W*_3_**	***W*_4_**	***W*_5_**	***W*_6_**	***W*_7_**
***Q*_1_**	(0.73, 0.07, 0.12)	(0.76, 0.12, 0.09)	(0.74, 0.12, 0.06)	(0.77, 0.13, 0.1)	(0.11, 0.06, 0.77)	(0.12, 0.09, 0.78)
***Q*_2_**	(0.81, 0.12, 0.06)	(0.85, 0.07, 0.07)	(0.82, 0.01, 0.1)	(0.68, 0.1, 0.08)	(0.02, 0.02, 0.8)	(0.06, 0.05, 0.82)
***Q*_3_**	(0.77, 0.1, 0.1)	(0.74, 0.08, 0.12)	(0.76, 0.12, 0.09)	(0.77, 0.1, 0.11)	(0.12, 0.07, 0.71)	(0.11, 0.09, 0.73)
***Q*_4_**	(0.75, 0.12, 0.07)	(0.72, 0.12, 0.09)	(0.74, 0.14, 0.11)	(0.79, 0.08, 0.08)	(0.14, 0.08, 0.75)	(0.14, 0.07, 0.71)
***Q*_5_**	(0.75, 0.08, 0.12)	(0.79, 0.06, 0.12)	(0.77, 0.05, 0.13)	(0.74, 0.13, 0.09)	(0.1, 0.14, 0.71)	(0.12, 0.11, 0.72)

**Table 4:** Decision matrix given by the fourth expert.

***W*_1_**	***W*_2_**	***W*_3_**	***W*_4_**	***W*_5_**	***W*_6_**	***W*_7_**
***Q*_1_**	(0.74, 0.08, 0.11)	(0.77, 0.13, 0.05)	(0.75, 0.07, 0.09)	(0.78, 0.06, 0.11)	(0.12, 0.04, 0.76)	(0.16, 0.07, 0.75)
***Q*_2_**	(0.84, 0.05, 0.05)	(0.82, 0.07, 0.1)	(0.85, 0.04, 0.06)	(0.77, 0.02, 0.1)	(0.1, 0.05, 0.83)	(0.07, 0.08, 0.85)
***Q*_3_**	(0.77, 0.05, 0.09)	(0.74, 0.02, 0.1)	(0.77, 0.05, 0.11)	(0.75, 0.02, 0.13)	(0.1, 0.07, 0.71)	(0.14, 0.06, 0.78)
***Q*_4_**	(0.74, 0.07, 0.09)	(0.8, 0.06, 0.15)	(0.77, 0.03, 0.12)	(0.78, 0.04, 0.09)	(0.14, 0.07, 0.71)	(0.15, 0.09, 0.7)
***Q*_5_**	(0.78, 0.06, 0.07)	(0.8, 0.03, 0.14)	(0.75, 0.04, 0.11)	(0.77, 0.03, 0.11)	(0.11, 0.06, 0.72)	(0.09, 0.09, 0.7)

The PF-CPT-TOPSIS method is used to select the best urban and rural residents' basic pension insurance systems.

**Step 1**. Normalize and integrate decision matrices, then we can acquire the integrated matrix that is listed in Table 5.

**Table 5:** The integrated and normalized decision matrix.

***W*_1_**	***W*_2_**	***W*_3_**	***W*_4_**	***W*_5_**	***W*_6_**	***W*_7_**
(0.749, 0.076, 0.111)	(0.745, 0.094, 0.108)	(0.748, 0.09, 0.097)	(0.776, 0.093, 0.107)	(0.773, 0.064, 0.109)	(0.765, 0.081, 0.132)	(0.749, 0.076, 0.111)
(0.816, 0.046, 0.073)	(0.841, 0.08, 0.067)	(0.805, 0.027, 0.068)	(0.719, 0.065, 0.083)	(0.806, 0.058, 0.055)	(0.817, 0.059, 0.067)	(0.816, 0.046, 0.073)
(0.771, 0.085, 0.101)	(0.74, 0.067, 0.12)	(0.768, 0.092, 0.1)	(0.786, 0.061, 0.12)	(0.724, 0.065, 0.126)	(0.741, 0.083, 0.114)	(0.771, 0.085, 0.101)
(0.762, 0.108, 0.085)	(0.766, 0.065, 0.117)	(0.755, 0.079, 0.121)	(0.758, 0.073, 0.106)	(0.749, 0.083, 0.132)	(0.708, 0.082, 0.129)	(0.762, 0.108, 0.085)
(0.751, 0.086, 0.103)	(0.762, 0.06, 0.119)	(0.737, 0.077, 0.118)	(0.758, 0.09, 0.112)	(0.739, 0.076, 0.121)	(0.73, 0.099, 0.098)	(0.751, 0.086, 0.103)

**Step 2**. Utilize the entropy method to calculate the original weights of all attributes.

**(1)** Calculate the average PFN of each attribute (see Table 6).

**Table 6:** The average PFN of each attribute.

***W*_1_**	***W*_2_**	***W*_3_**	***W*_4_**	***W*_5_**	***W*_6_**
(0.77, 0.08, 0.095)	(0.771, 0.073, 0.106)	(0.762, 0.073, 0.101)	(0.76, 0.076, 0.106)	(0.758, 0.069, 0.109)	(0.752, 0.081, 0.108)

**(2)** Obtain the numbers of Hamming distance by utilizing Eq (11), and the result is presented in Table 7.

**Table 7:** The numbers of Hamming distance.

	***W*_1_**	***W*_2_**	***W*_3_**	***W*_4_**	***W*_5_**	***W*_6_**
***Q*_1_**	0.017	0.02	0.013	0.013	0.01	0.012
***Q*_2_**	0.039	0.052	0.041	0.031	0.041	0.051
***Q*_3_**	0.003	0.023	0.007	0.022	0.024	0.008
***Q*_4_**	0.012	0.007	0.01	0.002	0.013	0.031
***Q*_5_**	0.014	0.011	0.02	0.005	0.015	0.019

**(3)** Acquire the original weights of all attributes (see Table 8).

**Table 8:** The original weights of all attributes.

	***W*_1_**	***W*_2_**	***W*_3_**	***W*_4_**	***W*_5_**	***W*_6_**
**i**	0.179	0.175	0.157	0.234	0.104	0.151

**Step 3**. Calculate all score numbers of PFNs in Table 5 (see Table 9).

**Table 9:** The score numbers of PFNs.

	***W*_1_**	***W*_2_**	***W*_3_**	***W*_4_**	***W*_5_**	***W*_6_**
***Q*_1_**	0.637	0.636	0.652	0.669	0.664	0.633
***Q*_2_**	0.743	0.775	0.737	0.636	0.75	0.75
***Q*_3_**	0.671	0.62	0.667	0.667	0.598	0.627
***Q*_4_**	0.676	0.649	0.634	0.652	0.617	0.579
***Q*_5_**	0.648	0.643	0.618	0.646	0.617	0.633

**Step 4**. Figure out the positive ideal point and negative ideal point of each attribute. The overcome is shown as follows:

Positive ideal points:


$$\begin{array}{l} s_{1}^{+}=\left(0.816,0.046,0.073\right)\;s_{2}^{+}=\left(0.841,0.08,0.067\right)\\ s_{3}^{+}=\left(0.805,0.027,0.068\right)\\ s_{4}^{+}=\left(0.776,0.093,0.107\right)\;s_{5}^{+}=\left(0.806,0.058,0.055\right)\\ s_{6}^{+}=\left(0.817,0.059,0.067\right) \end{array}$$

Negative ideal points:


$$\begin{array}{l} s_{1}^{-}=\left(0.749,0.076,0.111\right)\;s_{2}^{-}=\left(0.74,0.067,0.12\right)\\ s_{3}^{-}=\left(0.737,0.077,0.118\right)\;s_{4}^{-}=\left(0.719,0.065,0.083\right)\\ s_{5}^{-}=\left(0.724,0.065,0.083\right)\;s_{6}^{-}=\left(0.708,0.082,0.129\right) \end{array}$$

**Step 5**. Find out all Hamming distances between PFNs and positive ideal points (see Table 10).

**Table 10:** The Hamming distance between PFNs and positive ideal points.

	***W*_1_**	***W*_2_**	***W*_3_**	***W*_4_**	***W*_5_**	***W*_6_**
***Q*_1_**	0.054	0.069	0.052	0	0.033	0.049
***Q*_2_**	0	0	0	0.045	0	0
***Q*_3_**	0.04	0.074	0.042	0.015	0.064	0.06
***Q*_4_**	0.048	0.058	0.051	0.015	0.054	0.082
***Q*_5_**	0.053	0.062	0.061	0.012	0.057	0.066

**Step 6**. Find out all Hamming distances between PFNs and negative ideal points (see Table 11).

**Table 11:** The Hamming distance between PFNs and negative ideal points.

	***W*_1_**	***W*_2_**	***W*_3_**	***W*_4_**	***W*_5_**	***W*_6_**
***Q*_1_**	0	0.011	0.014	0.045	0.033	0.035
***Q*_2_**	0.054	0.074	0.061	0	0.064	0.082
***Q*_3_**	0.018	0	0.025	0.049	0	0.023
***Q*_4_**	0.019	0.016	0.012	0.03	0.019	0
***Q*_5_**	0.005	0.015	0	0.035	0.011	0.023

**Step 7**. Figure out the value of combined gains and losses.


$$\begin{array}{l} L_{1}^{+}=0.158\;L_{2}^{+}=0.317\;L_{3}^{+}=0.121\\ L_{4}^{+}=0.094\;L_{5}^{+}=0.103\\ L_{1}^{-}=-0.506\;L_{2}^{-}=-0.098\;L_{3}^{-}=-0.621\\ L_{4}^{-}=0.663\;L_{5}^{-}=-0.646 \end{array}$$

**Step 8**. Obtain the closeness coefficient δ_*d*_ of each alternative.

$$\begin{array}{l} \begin{array}{c} \begin{array}{c} \delta_{1}=-4.651\;\delta_{2}=0\;\delta_{3}=-5.938 \end{array}\;\delta_{4}=-6.451\;\delta_{5}=-6.243 \end{array} \end{array}$$

**Step 9**. Rank all companies in ascending order, and the overcome is listed as follows:

$$\begin{array}{l} Q_{2}>Q_{1}>Q_{3}>Q_{5}>Q_{4} \end{array}$$

We can see that *Q*_2_ is optimal urban and rural residents' basic pension insurance systems. For the maximum benefit, the *Q*_2_ urban and rural residents' basic pension insurance systems should be chosen to cooperate.

## Comparison analysis with some existing methods

For the sake of testing the validity of PT-CPT-TOPSIS approach, we compare the overcome of PT-CPT-TOPSIS approach with PFWA operator (37), PF-EDAS (picture fuzzy EDAS) approach (53), and PF-TODIM (picture fuzzy TODIM) approach (41) for the numerical instance in Section 5. In order to minimize errors, we still utilize the original weights of attributes *i* = (0.179, 0.175, 0.157, 0.234, 0.104, 0.151)^*T*^ and the previous weights of DMs *R*_*E*_1__ = 0.2, *R*_*E*_2__ = 0.3, *R*_*E*_3__ = 0.3, *R*_*E*_4__ = 0.2. The detailed steps are as follows:

### Compare with PFWA operator

In this portion, we tackle the problem of selecting an optimal urban and rural residents' basic pension insurance system by PFWA operator approach. The procedure of PFWA operator approach is shown as follows:

**Step 1**. Aggregate all PFNs of each company in Table 5 by PFWA operator function (see Table 12).

**Table 12:** The aggerating result of each company.

	**PFWA**
***Q*_1_**	(0.757, 0.083, 0.111)
***Q*_2_**	(0.81, 0.052, 0.07)
***Q*_3_**	(0.756, 0.077, 0.113)
***Q*_4_**	(0.751, 0.081, 0.115)
***Q*_5_**	(0.746, 0.08, 0.112)

**Step 2**. Figure out the score values of all aggregated PFNs.


$$\begin{array}{l} \begin{array}{c} \begin{array}{c} Y_{1}=0.646\;Y_{2}=0.74\;Y_{3}=0.643 \end{array}\;Y_{4}=0.636\;Y_{5}=0.634 \end{array} \end{array}$$

Ultimately, the ranking of all urban and rural residents' basic pension insurance system is shown as follows:


$$\begin{array}{l} Q_{2}>Q_{1}>Q_{3}>Q_{4}>Q_{5} \end{array}$$

### Compare with PF-EDAS method

In this portion, the PF-EDAS approach is used to tackle all PFSs in Table 5 to select an optimal urban and rural residents' basic pension insurance system. The procedure of PF-EDAS approach is listed as follows:

**Step 1**. Compute the average value of score numbers in Table 9 of all attributes (see Table 13).

**Table 13:** The average value of score numbers.

	***W*_1_**	***W*_2_**	***W*_3_**	***W*_4_**	***W*_5_**	***W*_6_**
${\bar{s}}^{*}$	0.675	0.665	0.662	0.654	0.649	0.644

**Step 2**. Find out the Positive Distance from Average (PDA) and Negative Distance from Average (NDA) by utilizing Eq (19) and Eq (20) (see Tables 14, 15).


(19) $$\begin{array}{l} PDA_{df}=\{\begin{array}{cc} H\left(s_{df}\right)-H\left({\bar{s}}_{f}\right), & \text{if }s_{df}>{\bar{s}}_{f}\\ 0, & \text{if }s_{df}\leq {\bar{s}}_{f} \end{array} \end{array}$$

(20) $$\begin{array}{l} NDA_{df}=\{\begin{array}{cc} H\left({\bar{s}}_{f}\right)-H\left(s_{df}\right), & \text{if }s_{df}<{\bar{s}}_{f}\\ 0, & \text{if }s_{df}\geq {\bar{s}}_{f} \end{array} \end{array}$$

**Table 14:** The PDA matrix.

	***W*_1_**	***W*_2_**	***W*_3_**	***W*_4_**	***W*_5_**	***W*_6_**
***Q*_1_**	0	0	0	0.023	0.023	0
***Q*_2_**	0.101	0.166	0.114	0	0.155	0.164
***Q*_3_**	0	0	0.008	0.02	0	0
***Q*_4_**	0.002	0	0	0	0	0
***Q*_5_**	0	0	0	0	0	0

**Table 15:** The NDA matrix.

	***W*_1_**	***W*_2_**	***W*_3_**	***W*_4_**	***W*_5_**	***W*_6_**
***Q*_1_**	0.056	0.042	0.015	0	0.023	0
***Q*_2_**	0	0	0	0.027	0155	0.164
***Q*_3_**	0.007	0.067	0	0	0	0
***Q*_4_**	0	0.024	0.042	0.003	0	0
***Q*_5_**	0.04	0.032	0.065	0.012	0	0

**Step 3**. Figure out the weighted normalized PDA and NDA by utilizing Eq (21, 22).

(21) $$\begin{array}{l} \begin{array}{c} NSP_{d}=\frac{\overset{h}{\underset{f=1}{\sum }}i_{f}PDA_{df}}{\underset{d}{\max}\left(\overset{h}{\underset{f=1}{\sum }}i_{f}PDA_{df}\right)}\;d=1,2,...,g \end{array} \end{array}$$

(22) $$\begin{array}{l} \begin{array}{c} NSN_{d}=1-\frac{\overset{h}{\underset{f=1}{\sum }}i_{f}NDA_{df}}{\underset{d}{\max}\left(\overset{h}{\underset{f=1}{\sum }}i_{f}NDA_{df}\right)}\;d=1,2,...,g \end{array} \end{array}$$

Then the overcome is described as follows:


$$\begin{array}{l} NSP_{1}=0.073\;NSP_{2}=1\;NSP_{3}=0.056\\ NSP_{4}=0.003\;NSP_{5}=0\;NSN_{1}=0.532\;NSN_{2}=0\\ NSN_{3}=0.726\;NSN_{4}=0.755\;NSN_{5}=0.452 \end{array}$$

**Step 4**. Obtain the appraisal score (*AS*) of each alternative by using Eq (23).

(23) $$\begin{array}{l} \begin{array}{c} AS_{d}=\frac{1}{2}\left(NSP_{d}+NSN_{d}\right)\;d=1,2,...,g \end{array} \end{array}$$

$$\begin{array}{l} AS_{1}=0.302AS_{2}=0.5AS_{3}=0.391AS_{4}=0.379\\ AS_{5}=0.226 \end{array}$$

According to the overcome of *AS*, the order is listed as follows:


$$\begin{array}{l} Q_{2}>Q_{3}>Q_{4}>Q_{1}>Q_{5} \end{array}$$

### Compare with PF-TODIM method

In this portion, we can assess the above alternative by utilizing the picture fuzzy TODIM (PF-TODIM) (41). The procedure of PF-TODIM approach is described as follows:

**Step 1**. Find out the relative weights of all attributes by using Eq (24).

(24) $$\begin{array}{l} \begin{array}{c} i_{f}^{*}=\frac{i_{f}}{\underset{f}{\max}\left(i_{f}\right)},\;f=1,2,...,h \end{array} \end{array}$$

The consequence of relative weights is shown as follows:


$$\begin{array}{l} i_{1}^{*}\;=\;0.765i_{2}^{*}\;=\;0.748i_{3}^{*}\;=\;0.671\;i_{4}^{*}\;=\;1\;i_{5}^{*}\;=\;0.444\\ i_{6}^{*}\;=\;0.654 \end{array}$$

**Step 2**. Calculate the dominance degree of the alternative *Q*_*d*_ over the alternative that represents the dominance degree of each alternative by using Eq (25) and Eq (26).


(25) $$\begin{array}{l} \begin{array}{c} \varsigma_{d}=\varsigma \left(s_{d},s_{\sigma }\right)=\overset{h}{\underset{f=1}{\sum }}\tau_{f}\left(s_{d},s_{\sigma }\right)\;d,\sigma =1,2,...,g \end{array} \end{array}$$

(26) $$\begin{array}{l} \tau_{f}\left(s_{d},s_{\sigma }\right)=\{\begin{array}{c} \begin{array}{cc} \sqrt{\frac{i_{f}^{*}e\left(s_{df},s_{\sigma f}\right)}{\overset{h}{\underset{f=1}{\sum }}i_{f}^{*}}} & \text{if }s_{df}>s_{\sigma f} \end{array}\\ \begin{array}{cc} 0, & \text{if }s_{df}=s_{\sigma f} \end{array}\\ \begin{array}{cc} -\frac{1}{\upsilon }\sqrt{\frac{e\left(s_{df},s_{\sigma f}\right)\overset{h}{\underset{f=1}{\sum }}i_{f}^{*}}{i_{f}^{*}}} & \text{if }s_{df}<s_{\sigma f} \end{array} \end{array} \end{array}$$

The overcome of dominance degree of each alternative is described as follows:


$$\begin{array}{l} \varsigma_{1}\;=\;-1.306\;\varsigma_{2}\;=\;1.12\;\varsigma_{3}\;=\;-2.266\;\varsigma_{4}\;=\;-2.297\\ \varsigma_{5}\;=\;-2.479 \end{array}$$

**Step 3**. Find out the numbers of overall dominance degree by using Eq (27).

(27) $$\begin{array}{l} \begin{array}{c} \xi_{d}=\frac{\varsigma_{d}-\min\varsigma_{d}}{\max\varsigma_{d}-\min\varsigma_{d}}\;d=1,2,...,g \end{array} \end{array}$$

Ultimately, we can obtain the result that is listed as follows:

$$\begin{array}{l} \begin{array}{c} \begin{array}{c} \xi_{1}=0.326\;\xi_{2}=1\;\xi_{3}=0.059 \end{array}\;\xi_{4}=0.051\;\xi_{5}=0 \end{array} \end{array}$$

The final ranking of all urban and rural residents' basic pension insurance system is *Q*_2_ > *Q*_1_ > *Q*_3_ > *Q*_4_ > *Q*_5_.

### Contrastive analysis

By coping the above issue with various approaches, we catalog overcomes in Table 16. Based on the table, we select *Q*_2_ company as an optimal partner without doubt, as shown in Table 16. Through the overcome, we can testify the accuracy of the novel approach. This verifies the PF-CPT-TOPSIS method is effective. These four given methods have their given advantages: (1) the PFWA operator emphasizes group decision influences; (2) the PF-EDAS method requires fewer computations, although it results in the same ranking of alternatives. The evaluation of alternatives in EDAS is based on distance measures from the average solutions in terms of each criterion unlike TOPSIS and VIKOR. (3) PF-TODIM method is an interactive MADM method according to the psychological behavior of decision-makers; and (4) the PF-CPT-TOPSIS method considers the benefits and losses of psychological activities that can make the decision more accurate than before.

**Table 16:** The contrastive analysis.

	**Sequence**
PF-CPT-TOPSIS	
PFWA operator	*Q*_2_ > *Q*_1_ > *Q*_3_ > *Q*_4_ > *Q*_5_
PF-EDAS approach	*Q*_2_ > *Q*_3_ > *Q*_4_ > *Q*_1_ > *Q*_5_
PF-TODIM approach	*Q*_2_ > *Q*_1_ > *Q*_3_ > *Q*_4_ > *Q*_5_

## Conclusion

Public policy is an important tool for the government to solve public problems, implement public management, improve public welfare, and achieve long-term national development and long-term stability. As an important link in the process of public policy analysis, public policy evaluation will have a greater impact on the results of the policy implementation process and the generation of new policies. As a public policy, the operation efficiency of urban and rural residential insurance determines whether the policy objectives can be achieved or not, and also determines the success or failure of the system. It is necessary to construct a scientific and reasonable evaluation framework and evaluation method to objectively display the operation efficiency of urban and rural residential insurance, which is conducive to discovering various problems in it, and provides a corresponding reference for the further improvement of urban and rural residential insurance in the future. In addition, urban and rural residential security is a livelihood project, which is closely related to the interests of urban and rural residents, and is not a simple redistribution issue. Moreover, from the perspective of resource scarcity, the input–output efficiency of the urban and rural residential insurance system should also be measured and evaluated to see whether the system can achieve the maximum output under the given input resources and the goal of sustainable development. Since the implementation of the combination of urban and rural residential insurance, how are the implementation status and operational efficiency of the system? What factors have a significant impact on the efficiency of system operation? How to promote the improvement of the operation efficiency of the urban and rural residential insurance system so as to achieve the goal of system sustainability? This is the research focus of this paper. Clarifying the above problems will help us to clearly find the errors in the policy design and actual operation of the urban and rural residential insurance system. It is of great significance to further improve the system and achieve the goal of “supporting the elderly” for urban and rural residents, which is an important practical significance. In the manuscript, we put forward a novel model by combining the original TOPSIS method with CPT under a PFS environment. In the traditional TOPSIS approach, we usually select the optimal alternative by comparing the distance between each alternative with an ideal point or a negative ideal point. However, we ignore the DMs' psychological factors. We combine CPT with TOPSIS to make up for the disadvantage. In the procedure of the novel model, we take advantage of the entropy method to calculate the original weights of attributes. For testifying the effectiveness and validity of the new approach, we give an instance for operational efficiency evaluation of urban and rural residents' basic pension insurance systems. Ultimately, we compare the overcome of the PF-CPT-TOPSIS approach with other methods. Taking DMs' psychological factors into consideration can help us make better use of decision information. Thus, the main contributions of this study are as follows: (1) the TOPSIS method is extended by PFSs with unknown weight information; (2) the entropy method is used to obtain the original weights of attributes; (3) the PF-CPT-TOPSIS method is used to deal with the MAGDM problems under PFSs; (4) a numerical instance for operational efficiency evaluation of urban and rural residents' basic pension insurance systems is proposed to testify the effectiveness of new method; and (5) some comparative studies are provided to give effect to the rationality of PF-CPT-TOPSIS approach.

Further research should focus on the following some meaningful issues in our future studies: (1) The objective of this paper is mainly to analyze the situation that the reference point has been given by the decision maker, but there are still unknown reference points in real life, which requires the decision maker to select the reference point according to a certain method. Selection is an important research direction of prospect theory. (2) In our future works, we shall use Criteria Importance Though Intercrieria Correlation (CRITIC) (54), Method Based on the Removal Effects of Criteria (MEREC) (55, 56), or subjective methods like Full Consistency Method (FUCOM), Level-Based Weight Assessment (LBWA), or Best–Worst Method (BWM) (57–61) instead of Entropy method. (3) At the same time, how to predict the future development direction of the scheme according to prospect theory is also the focus of future research. The proposed decision-making method should also be applied to actual cases, and consider whether the proposed method is still effective under extreme conditions. For the state displayed by each attribute in different schemes, how to summarize and predict this under the existing conditions, so as to ensure that the decision-making results are more forward-looking is also an urgent problem to be studied. (4) The MADM problem is a decision-making problem encountered by all walks of life in today's society, ranging from product selection to project evaluation. It can retain the subjective preferences of decision-makers while being objective, but there is still a lot of research space for applying prospect theory to solve MADM problems. (5) The CPT-TOPSIS approach will be widely used in other uncertain and fuzzy settings in the future (62–68).

## Data availability statement

The data used to support the findings of this study are included in the article.

## Author contributions

The author confirms being the sole contributor of this work and has approved it for publication.

## Conflict of interest

The author declares that the research was conducted in the absence of any commercial or financial relationships that could be construed as a potential conflict of interest.

## Publisher's note

All claims expressed in this article are solely those of the authors and do not necessarily represent those of their affiliated organizations, or those of the publisher, the editors and the reviewers. Any product that may be evaluated in this article, or claim that may be made by its manufacturer, is not guaranteed or endorsed by the publisher.
